# Fig Meal Replacement Powder Ameliorates Obesity, Oxidative Stress and Intestinal Microbiota in Mice Fed With High‐Fat Diet

**DOI:** 10.1002/fsn3.70104

**Published:** 2025-04-20

**Authors:** Mingze Xu, Longfei Zhang, Xiaoxiao Liu, Yigu Tian, Bingkui Wang, Tianzhu Guan, Wenliong Ma, Hengxian Qu, Dawei Chen, Lixia Xiao

**Affiliations:** ^1^ School of Food Science and Engineering Yangzhou University Yangzhou China; ^2^ Key Laboratory of Dairy Biotechnology and Safety Control Yangzhou China

**Keywords:** antioxidant, gut microbiota, meal replacement powder, obesity

## Abstract

Figs, known for their high‐antioxidant capacity, have shown potential in regulating obesity. However, research on fig‐based products and the mechanisms behind their effects remains limited. This study aims to systematically evaluate the potential of fig meal replacement powder (FMRP) in regulating obesity and mitigating obesity‐induced oxidative stress through both in vitro and in vivo experiments, while also elucidating its underlying mechanisms. The results demonstrated that FMRP exhibited superior nutritional value and antioxidant activity compared to commercially available alternatives. Furthermore, FMRP significantly reduced weight gain, improved lipid metabolism, alleviated liver damage and oxidative stress, and positively modulated the gut microbiota in high‐fat diet (HFD)‐fed mice. Gut microbiota analysis showed that FMRP could restore the gut microbiota of hfd mice. For instance, it reduced the *Firmicutes*/*Bacteroidetes* (F/B) ratio. The correlation analysis has revealed the key bacterial genera related to obesity and oxidative stress. The key bacterial genera related to obesity include *Desulfovibrio*, *Lachnoclostridium*, etc., while the key bacterial genera related to oxidative stress include *Bifidobacterium*, *Lactobacillus*, and *Turicibacter*, etc. In conclusion, FMRP effectively alleviates oxidative stress, improves lipid metabolism, and modulates the gut microbiota, highlighting its potential as a functional food for obesity management.

## Graphical abstract

This study aims to investigate the effects of an optimized high‐antioxidant fig meal replacement powder on obesity‐related traits in a high‐fat diet‐induced obese mouse model. The study will evaluate the antioxidant and immune‐modulating properties of the FMRP in vivo. Additionally, by analyzing and comparing the gut microbiota, it will assess the powder's impact on the structure of the gut microbiota and explore the relationship between gut microbiota composition and obesity traits.

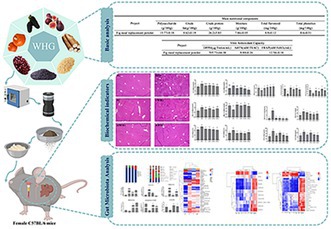

## 1 Introduction

Obesity, a metabolic disorder caused by a prolonged imbalance between energy intake and expenditure, has emerged as a significant global public health challenge. It is a major risk factor for metabolic diseases, including diabetes, fatty liver disease, cardiovascular disorders, and hyperglycemia (Katta et al. 2021; Prendergast et al. 2022). Chronic obesity leads to excessive lipid accumulation and adipose tissue expansion, resulting in elevated levels of reactive oxygen species (ROS) and exacerbated oxidative stress (Swiatkiewicz et al. 2023). The hypertrophy of adipose tissue further promotes the secretion of pro‐inflammatory cytokines, such as interleukin‐6 (IL‐6) and tumor necrosis factor‐alpha (TNF‐α), contributing to chronic low‐grade inflammation (Gregor and Hotamisligil 2011). TNF‐α interacts with specific receptors to activate the NF‐κB signaling pathway, enhancing ROS production and intensifying oxidative stress, ultimately leading to systemic damage (Chandel et al. 2001).

Traditional weight‐loss strategies, such as bariatric surgery and pharmacotherapy, often come with undesirable side effects, limiting their widespread application (Son 2024). In contrast, meal replacement powders, formulated from high‐quality fruits, vegetables, and grains, provide an effective dietary intervention with benefits including low caloric intake, a slow glycemic response, and enhanced satiety, making them increasingly popular for obesity management (Volpe et al. 2016).

Among functional food components, anthocyanins have been shown to enhance hepatic antioxidant enzyme activity (Solomon et al. 2006), including superoxide dismutase (SOD) and glutathione peroxidase (GSH‐PX), while reducing malondialdehyde (MDA) levels, thereby mitigating oxidative stress (Wu et al. 2016). Polysaccharides derived from figs regulate the expression of inflammatory cytokines such as TNF‐α and IL‐6 and stimulate macrophage activity both in vivo and in vitro (Ye et al. 2024). Additionally, Zhao et al. (2020) demonstrated that fermented figs significantly improved gut microbiota composition, enhanced immune responses, and restored organ function, ultimately contributing to weight reduction in immunodeficient mice (Wu et al. 2024).

Building upon this foundation, our previous research developed a FMRP with high‐antioxidant capacity and potential anti‐obesity effects. However, the mechanisms by which gut microbiota modulate obesity and oxidative stress‐related liver damage remain unclear and require further investigation.

## 2 Materials and Methods

### 2.1 Reagents and Materials

Dried figs were sourced from Nantong, Jiangsu Province, China. Commercial meal replacement powder, mulberry powder, roasted adzuki bean powder, roasted black sesame powder, roasted brown rice powder, pumpkin powder, and roasted yam powder were obtained from Yonghui Supermarket in Yangzhou, China. Reagents such as methanol, concentrated sulfuric acid, phenol, glucose, and trichloroacetic acid were supplied by Shanghai Hushi Laboratory Equipment Co. Ltd.

### 2.2 Sample Preparation and Nutritional Composition Analysis

Dried figs with sufficient dryness and no mold were selected and pulverized using a HS0965 wall‐breaking machine (Westinghouse Electric Corporation, USA). The resulting powder was sieved through an 80‐mesh screen and stored in sealed bags. The plant‐based ingredient powders were mixed in the following mass ratio: fig: mulberry: yam: adzuki bean: brown rice: pumpkin: black sesame = 7:4:3:3:3:2:1. After thorough mixing, the blend was stored at 4°C in sealed bags.

For preparing the FMRP solution, the powder was mixed with hot water (90°C) at a 1:10 ratio and stirred until smooth, ensuring no visible lumps. The solution was set aside for further experiments. The contents of total polysaccharides, crude protein, crude fat, moisture, total polyphenols, and total flavonoids were determined using the Phenol‐sulfuric acid method, Coomassie brilliant blue method, Soxhlet extraction method, Drying loss method, Folin–Ciocalteu method, and Aluminum nitrate colorimetric method, respectively (Lin et al. 2023).

### 2.3 Determination of Physicochemical Properties

Take 5 g (m_1_) of FMRP sample and mix thoroughly with distilled water at a ratio of 1:10 (m/v) (m_2_). Place the mixture in a 60°C water bath and continuously stir while heating. After 30 min, remove the mixture and centrifuge at 1800 × g for 15 min (Kusumayanti et al. 2015). The swelling power (SP) is calculated as follows:

$$\mathrm{SP}\;\left(g/g\right)=m_{2}/m_{1}$$

Take 5 g (m_1_) of FMRP sample and mix thoroughly with deionized water at a ratio of 1:20 (m/v) (m_2_). Place the mixture in a 25°C water bath for 24 h, then centrifuge at 2300 × g for 10 min. Discard the supernatant and record the weight of the remaining particles (Shen et al. 2020). The water‐holding capacity (WHC) is calculated as follows:

$$\mathrm{WHC}\;\left(g/g\right)=\left(m_{2}-m_{1}\right)/m_{1}$$

Take 1 g (m) of FMRP sample and dissolve it in 30 mL of distilled water. Place the mixture in a 30°C water bath for 30 min, shake once, then shake for 5 min, and centrifuge at 15000 × g for 30 min (Heo et al. 2014). Weigh sediment mass after centrifugation (m_1_) and dry the supernatant (m_2_). The water absorption index (WAI) and water solubility index (WSI) are calculated using the following formulas:

$$\mathrm{WAI}\;\left(\%\right)=\left(m_{1}/m\right)\times 100\%$$

$$\mathrm{WSI}\;\left(\%\right)=\left(m_{2}/m\right)\times 100\%$$

### 2.4 Determination of Antioxidant Capacity In Vitro

The DPPH radical scavenging activity, ABTS radical scavenging activity, and Fe^2+^ scavenging activity of the FMRP solution were measured according to the instructions provided by the DPPH radical scavenging assay kit, total antioxidant capacity (T‐AOC) assay kit (ABTS, FRAP, Nanjing Jiancheng Bioengineering Institute, Nanjing, China). Trolox standard powder (Nanjing Jiancheng Bioengineering Institute, Nanjing, China) was dissolved in 80% methanol and diluted to various concentrations to generate the DPPH calibration curve. The 10 mM Trolox solution was similarly diluted to different concentrations to create the ABTS and FRAP calibration curves.

### 2.5 Animals and Experimental Design

Female SPF C57BL/6J mice (18 ± 0.20 g, 5 weeks old) were obtained from Jiangsu Wukong Biotechnology Co. Ltd. (License No: SCXK‐2022‐0006, Nanjing, China). The study protocol was approved by the Ethics Committee on Experimental Animals at Yangzhou University (202501026). All mice were housed in the Experimental Animal Facility under controlled conditions (temperature: 26°C ± 1°C, humidity: 55% ± 5%) with a 12‐h light/dark cycle and were given *ad libitum* access to food and water. The feed (product number: XT93M) was supplied by Jiangsu Synergy Pharmaceutical Bioengineering Co. Ltd. (Nanjing, China).

After a 7‐day acclimatization period, 42 mice were randomly divided into six groups (7 mice per group): normal diet control (NC) group, HFD‐induced obesity (HFD) group, positive control (PC) group, low‐dose FMRP (LWHG) group, medium‐dose FMRP (MWHG) group, and high‐dose FMRP (HWHG) group.

The NC group was fed a normal diet, while all other groups were provided a hfd to induce obesity. After 4 weeks, the NC and HFD groups received physiological saline via gavage. The PC group was administered a commercially available FMRP solution (0.5 mg/g/day) by gavage, while the LWHG, MWHG, and HWHG groups received FMRP solutions at doses of 0.25 mg/g/day, 0.5 mg/g/day, and 1 mg/g/day, respectively. The intervention lasted 4 weeks, with daily food intake recorded and body weight measured weekly.

### 2.6 Sample Collection

After intervention, the mice were fasted and underwent water restriction for 12 h. Following ether anesthesia, blood was collected from the eyeballs, and the mice were euthanized by cervical dislocation. The collected blood was allowed to stand at 37°C for 10 min, then centrifuged at 3000 × g at 4°C for 10 min to obtain serum, which was subsequently stored at −80°C. The liver was dissected, weighed, and rinsed with PBS solution. A portion of the liver was homogenized in PBS solution (1:9, w/v) using a tissue homogenizer to prepare the liver homogenate. The homogenate was centrifuged at 1800 × g at 4°C for 10 min, and the supernatant was collected for further analysis. Fresh cecal contents were also collected in 1.5 mL cryogenic tubes and stored at −80°C.

### 2.7 Biochemical Analysis

An automatic biochemical analyzer (7020, HITACHI, Japan) was used to measure total cholesterol (TC), triglycerides (TG), low‐density lipoprotein cholesterol (LDL‐C), high‐density lipoprotein cholesterol (HDL‐C), alanine aminotransferase (ALT), and aspartate aminotransferase (AST) levels in both serum and liver homogenate samples. IL‐6 and TNF‐α levels were determined according to the instructions provided by the reagent kits (Shanghai Hualan Biological Technology Co. Ltd., Shanghai, China). Additionally, the activities of SOD, catalase (CAT), and GSH‐PX, as well as the contents of reduced glutathione (GSH) and MDA, were measured using assay kits from Nanjing Jiancheng Bioengineering Institute (Nanjing, China).

### 2.8 Preparation of Liver Tissue Sections

The livers were fixed with 4% paraformaldehyde, embedded in paraffin, and cut into 4 μm thick sections using a microtome (RM2255, Leica, Germany). The fixed slices were dewaxed with xylene, hydrated with gradient ethanol, and soaked in distilled water. Then the slices were stained with Hematoxylin and eosin (H&E) staining solution (Beijing Solarbio Science & Technology Co. Ltd., Beijing, China). After staining, the slices were soaked in ethanol and xylene sequentially for dehydration. The morphological changes of the livers of mice in each group could be examined under an optical microscope at 100× magnification to evaluate pathological differences in liver tissue across the groups.

### 2.9 Gut Microbiota Analysis

Colon content samples were randomly collected from the NC, HFD, PC, and MWHG groups. DNA extraction, PCR amplification, Illumina MiSeq sequencing, and post‐processing were carried out as described in (Zhang, Liu et al. 2024). Data analysis was performed using the I‐sanger platform (http://www.i‐sanger.com) provided by Majorbio.

### 2.10 Statistical Analyses

All experimental data are presented as mean ± standard deviation (SD) and were analyzed using one‐way analysis of variance (ANOVA) with SPSS 29.0. For comparisons involving three or more groups, one‐way ANOVA followed by Tukey's multiple comparison test was conducted. Differences were considered statistically significant at *p* < 0.05 and were indicated by different lowercase letters. Each parameter was measured at least three times. Graphs depicting group differences were created using GraphPad Prism 10.0 software.

## 3 Results and Discussion

### 3.1 Ingredients and Properties of FMRP

This study compared the nutritional components and physicochemical properties of FMRP with those of a commercially available meal replacement powder, as shown in Table 1. The FMRP contained significantly higher levels of polysaccharides, crude protein, total flavonoids, and total polyphenols compared to the commercial powder (*p* < 0.05), while its crude fat and moisture content were significantly lower (*p* < 0.05).

**TABLE 1:** Overview of FMRP Composition and Physicochemical Properties.

Project		FMRP	Commercially available meal replacement powder
Main nutritional components	Polysaccharide (g/100 g)	15.77 ± 0.18^b^	15.38 ± 0.28^a^
	Crude fat (g/100 g)	4.62 ± 0.19^a^	5.51 ± 0.22^b^
	Crude protein (g/100 g)	26.2 ± 5.83^b^	11.07 ± 0.58^a^
	Moisture (g/100 g)	7.86 ± 0.05^a^	8.55 ± 0.07^b^
	Total flavonoid (mg/100 g)	4.9 ± 0.12^b^	2.6 ± 0.14^a^
	Total phenolics (mg/100 g)	8.6 ± 0.51^b^	4.5 ± 0.90^a^
Physicochemical properties	SP (g/g)	1.51 ± 0.03^a^	1.43 ± 0.06^a^
	WHC (mL/mL)	4.52 ± 0.08^b^	1.72 ± 0.25^a^
	WAI (%)	27.91 ± 0.72^a^	29.47 ± 1.34^b^
	WSI (%)	499.35 ± 8.53^a^	526.24 ± 13.84^b^

*Note:* Different letters indicate significant differences (*P* < 0.05).

In terms of physicochemical properties, no significant difference was observed in the SP value between FMRP and the commercial powder (*p* > 0.05). However, the FMRP demonstrated a significantly higher WHC value (*p* < 0.05), indicating improved water‐holding capacity. Conversely, the WAI and WSI values of FMRP were significantly lower than those of the commercial powder (*p* < 0.05), suggesting enhanced stability and solubility of the fig‐based formulation.

### 3.2 Evaluation of the In Vitro Antioxidant Capacity of FMRP

The in vitro antioxidant capacity of the FMRP was assessed. The standard curve is presented in Figure S1, and the in vitro antioxidant results are summarized in Table 2. The FMRP exhibited significantly higher DPPH radical scavenging activity, ABTS radical scavenging activity, and Fe^2+^ chelating ability compared to the commercially available meal replacement powder (*p* < 0.05), with antioxidant capacities being 1.90, 1.98, and 2.47 times greater, respectively. These findings suggest that the FMRP possesses strong antioxidant properties.

**TABLE 2:** In Vitro Antioxidant Activity of FMRP Compared to Commercial Meal Replacement Powder.

	Measurement indicators		
	DPPH (μg Trolox/mL)	ABTS (mM TEAC)	FRAP (mM TEAC)
FMRP	705.73 ± 66.38^b^	9.89 ± 0.26^b^	0.95 ± 0.05^b^
Commercially available meal replacement powder	369.96 ± 43.28^a^	4.98 ± 0.06^a^	0.64 ± 0.05^a^

*Note:* Different letters indicate significant differences (*p* < 0.05).

### 3.3 Effects of FMRP on Basic Indicators in Obese Mice

**FIGURE 1:**
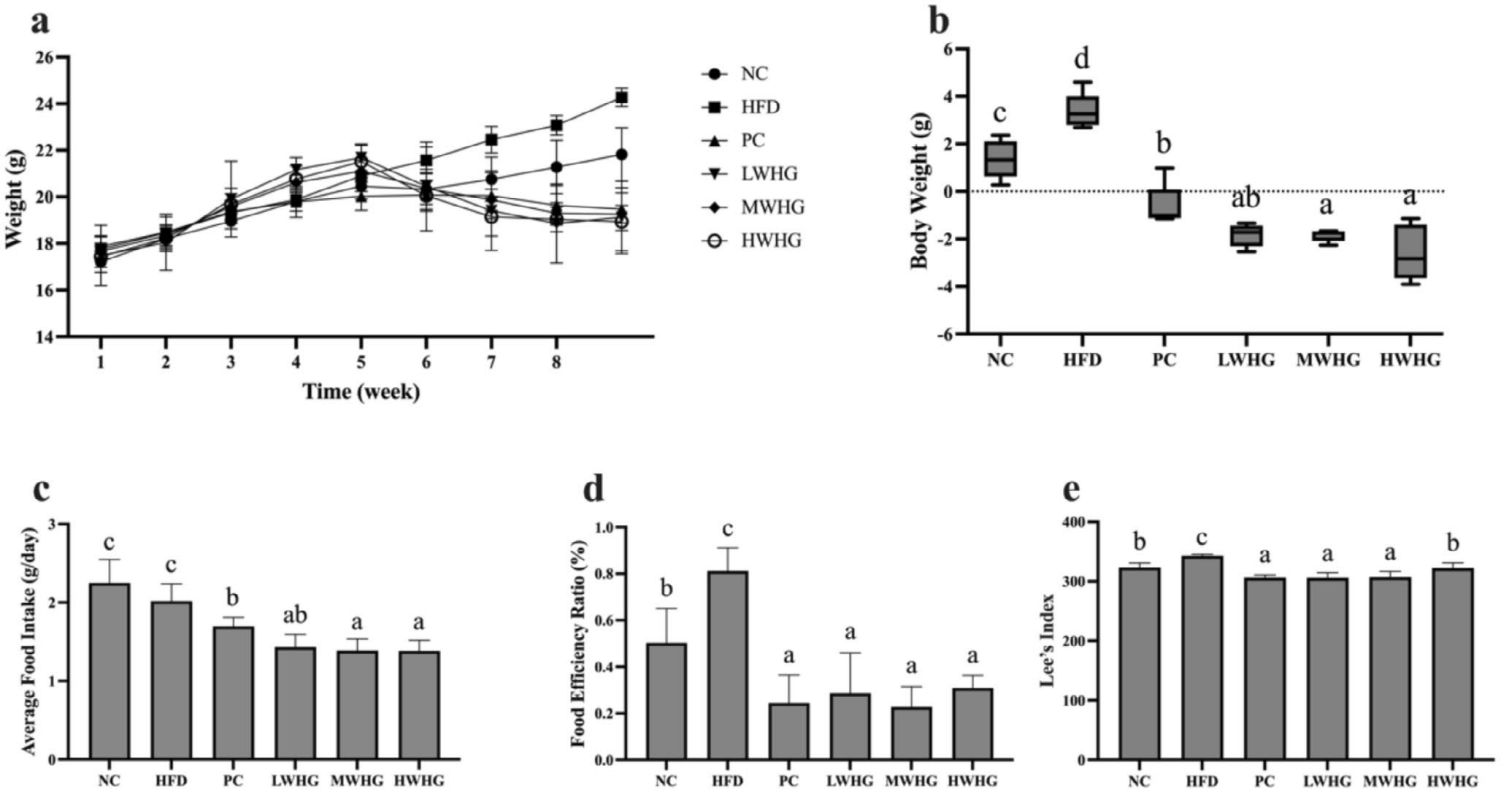
Effects of FMRP on Basic Indicators in Obese Mice (a) Dynamic body weight change curve, (b) Body weight variation in mice, (c) Average food intake, (d) Energy conversion efficiency, and (e) Lee's index. All data are expressed as mean ± standard deviation (*n* = 7). Different letters above the bars indicate statistically significant differences (*p* < 0.05), which also apply to the subsequent figures.

The effects of the FMRP on basic indicators in obese mice are presented in Figure 1, including weight variation, average food intake, energy conversion efficiency, and Lee's index. The results demonstrated that high, medium, and low doses of the FMRP inhibited weight gain induced by the HFD. The weight variation in the HFD group was significantly higher than in the other groups (*p* < 0.05), while the weight variation was significantly reduced following intervention with the FMRP (*p* < 0.05). Although no significant differences were observed among the different dosage groups (*p* > 0.05), all groups exhibited significantly lower weight variation compared to the HFD group (*p* < 0.05). Moreover, the FMRP significantly reduced food intake, the food efficiency ratio, and Lee's index (*p* < 0.05), with no significant differences between the MWHG and LWHG groups (*p* > 0.05). These findings suggest that the FMRP effectively suppresses appetite, reduces the food efficiency ratio, and helps regulate weight gain induced by a hfd.

### 3.4 Effects of FMRP on Blood Lipids and Liver in Mice

**FIGURE 2:**
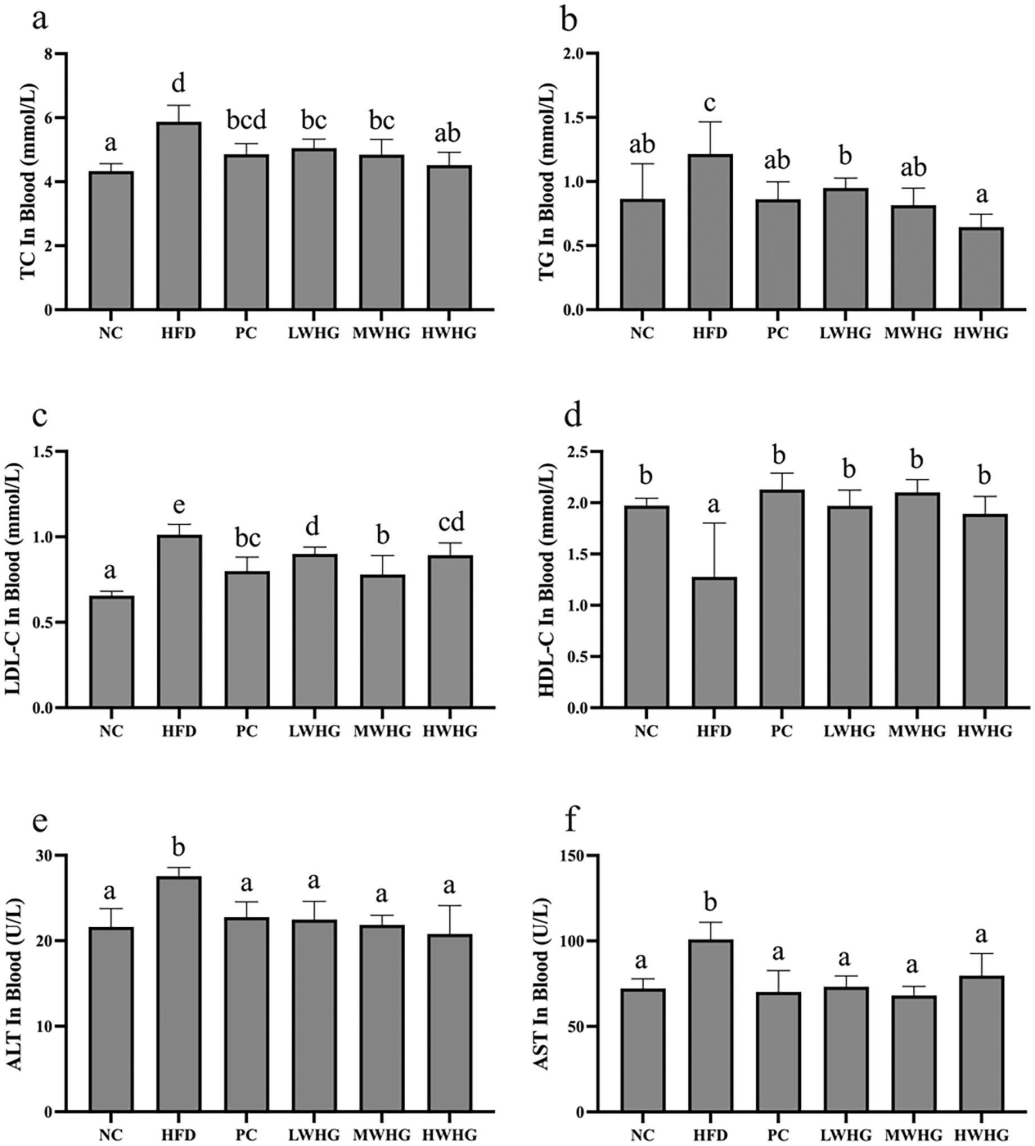
Effects of FMRP on Blood Lipids and Liver Function in Mice(a) TC levels, (b) TG levels, (c) LDL‐C levels, (d) HDL‐C levels, (e) ALT levels, and (f) AST levels. Different letters indicate significant differences at the *p* < 0.05 level for each column.

The effects of FMRP on blood lipid levels and liver damage in obese mice are presented in Figure 2, including measurements of TC, TG, LDL‐C, HDL‐C, ALT, and AST. The results showed that TC, TG, and LDL‐C levels were significantly higher in the HFD group compared to the NC group (*p* < 0.05). After intervention with FMRP, all dosage groups exhibited significantly lower levels of these markers compared to the HFD group (*p* < 0.05). Furthermore, the intervention significantly increased HDL‐C levels in the HFD group (*p* < 0.05), restoring them to levels comparable to the NC group (*p* > 0.05). No significant differences were observed among the different dosage groups (*p* > 0.05). These findings suggest that FMRP may effectively ameliorate lipid metabolism disorders induced by a hfd, with the medium dose showing the most pronounced regulatory effect. Additionally, ALT and AST levels, which were significantly elevated in the HFD group (*p* < 0.05), indicating liver damage after 8 weeks of hfd feeding, were significantly reduced in all dosage groups following intervention (*p* < 0.05), with no significant difference compared to the NC group (*p* > 0.05).

### 3.5 Effects of FMRP on Lipid Accumulation in the Liver of Mice

**FIGURE 3:**
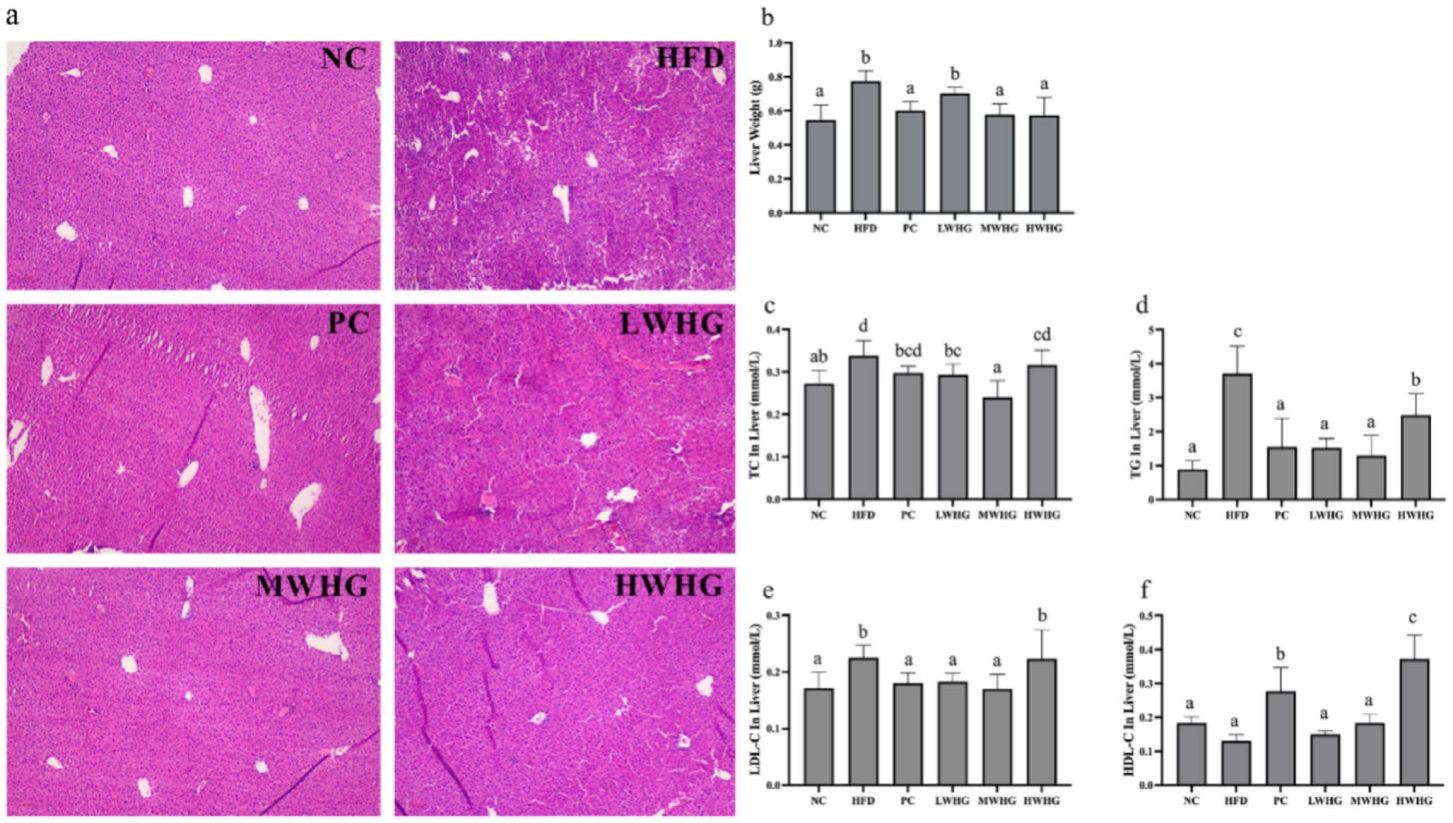
Effects of FMRP on Liver Lipid Accumulation in Mice (a) Liver tissue sections (×100), (b) Liver weight, (c) TC content in the liver, (d) TG content in the liver, (e) LDL‐C content in the liver, and (f) HDL‐C content in the liver. Different letters indicate significant differences at the *p* < 0.05 level for each column.

The effects of FMRP on liver lipid accumulation in obese mice are illustrated in Figure 3. As shown in Figure 3a, a large number of lipid vacuoles were present in the liver of mice in the HFD group compared to the NC group. However, following intervention with PC, LWHG, MWHG, and HWHG, the number of lipid vacuoles was significantly reduced. Notably, the MWHG and HWHG groups exhibited fewer lipid vacuoles than the LWHG group, suggesting that the hfd led to excessive lipid accumulation in the liver, overwhelming its metabolic capacity and resulting in increased liver weight. Figure 3b shows that liver weight in the HFD group was significantly higher (*p* < 0.05). After intervention with the FMRP, liver weight in all groups, except the LWHG group, was significantly reduced (*p* < 0.05). The lipid content in liver tissue, including TC, TG, LDL‐C, and HDL‐C, was also assessed. The results demonstrated that low and medium doses of FMRP significantly reduced TC levels in the liver (*p* < 0.05), while all doses significantly reduced TG levels (*p* < 0.05). Additionally, low and medium doses significantly reduced LDL‐C levels in the liver (*p* < 0.05), while only the HWHG group significantly increased HDL‐C levels (*p* < 0.05). These findings suggest that FMRP has the potential to regulate lipid metabolism disorders and abnormal lipid accumulation in the liver of obese mice.

### 3.6 Effects of FMRP on Oxidative Stress Damage and Inflammatory Response in Mice

**FIGURE 4:**
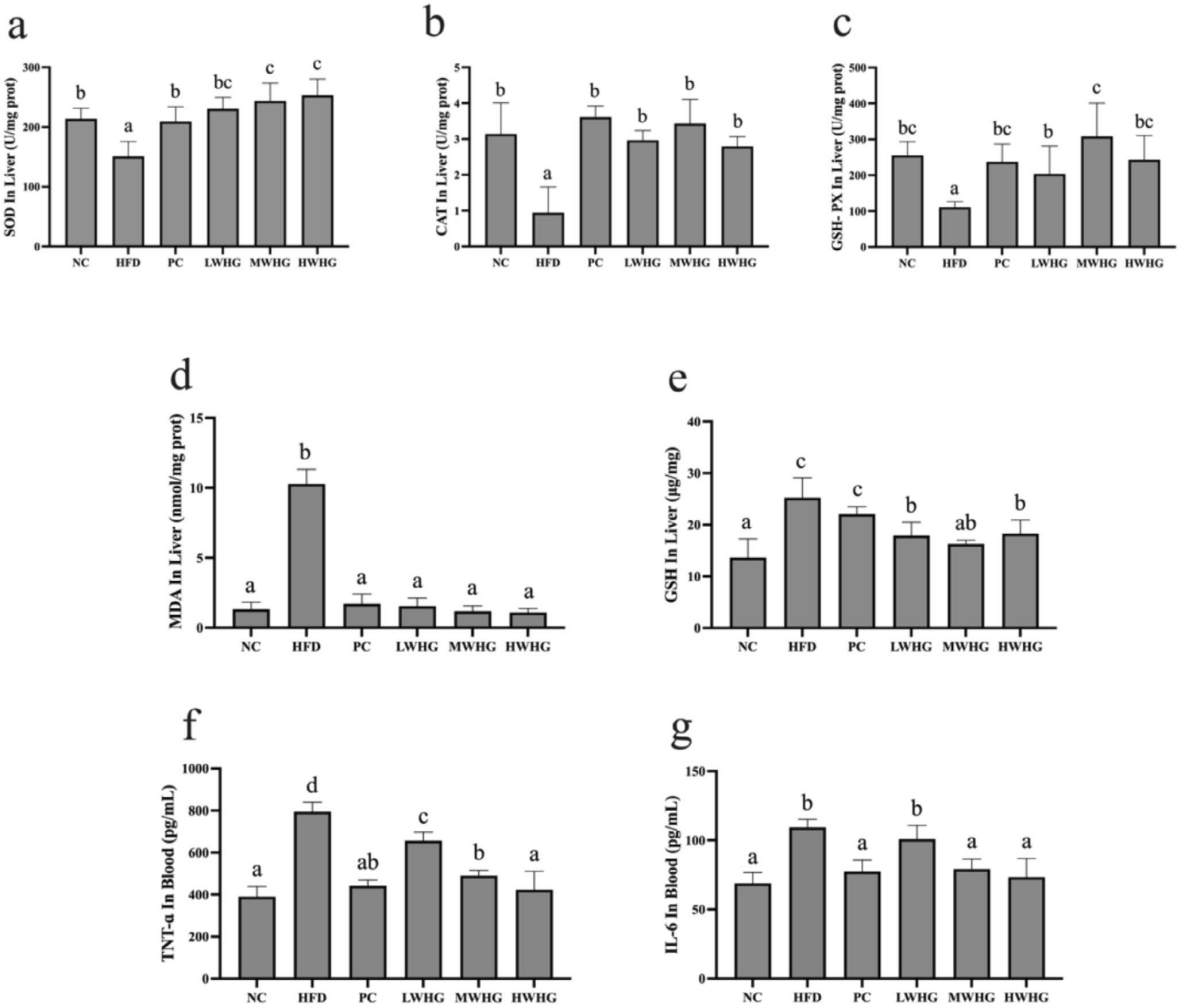
Effects of FMRP on Oxidative Stress Damage and Inflammatory Response in Mice (a) SOD enzyme activity, (b) CAT enzyme activity, (c) GSH‐PX content, (d) MDA content in the liver, (e) GSH content in the liver, (f) TNF‐α content in the blood, and (g) IL‐6 content in the blood. Different letters indicates significant differences at the *p* < 0.05 level for each column.

Obesity can suppress the body's antioxidant capacity, leading to oxidative stress damage and, over time, chronic low‐grade inflammation. The oxidative stress levels and inflammatory responses in mice were measured, and the results are shown in Figure 4. The findings indicated that a hfd significantly decreased the enzyme activities of SOD, CAT, and GSH‐PX in mice (*p* < 0.05). Following intervention with FMRP, enzyme activities in all dosage groups were significantly higher than those in the HFD group (*p* < 0.05). Among these, GSH‐PX enzyme activity in the MWHG and HWHG groups was significantly higher than in the LWHG group (*p* < 0.05), whereas no significant differences in CAT and SOD enzyme activities were observed between the LWHG, MWHG, and HWHG groups (*p* > 0.05). MDA and GSH are key metabolic products of oxidative stress. The levels of MDA and GSH in the liver were measured, as shown in Figure 4d,f. The results revealed that all dosages of FMRP (low, medium, and high) significantly reduced MDA and GSH levels in obese mice (*p* < 0.05), indicating a marked effect on reducing lipid peroxidation and enhancing antioxidant capacity in the liver. Additionally, the FMRP significantly lowered the levels of pro‐inflammatory factors IL‐6 (Figure 4f) and TNF‐α (Figure 4g) in obese mice (*p* < 0.05). These findings suggest that FMRP has the potential to regulate oxidative damage and inflammatory responses induced by obesity.

### 3.7 Effects of FMRP on Gut Microbiota Diversity in Mice

**FIGURE 5:**
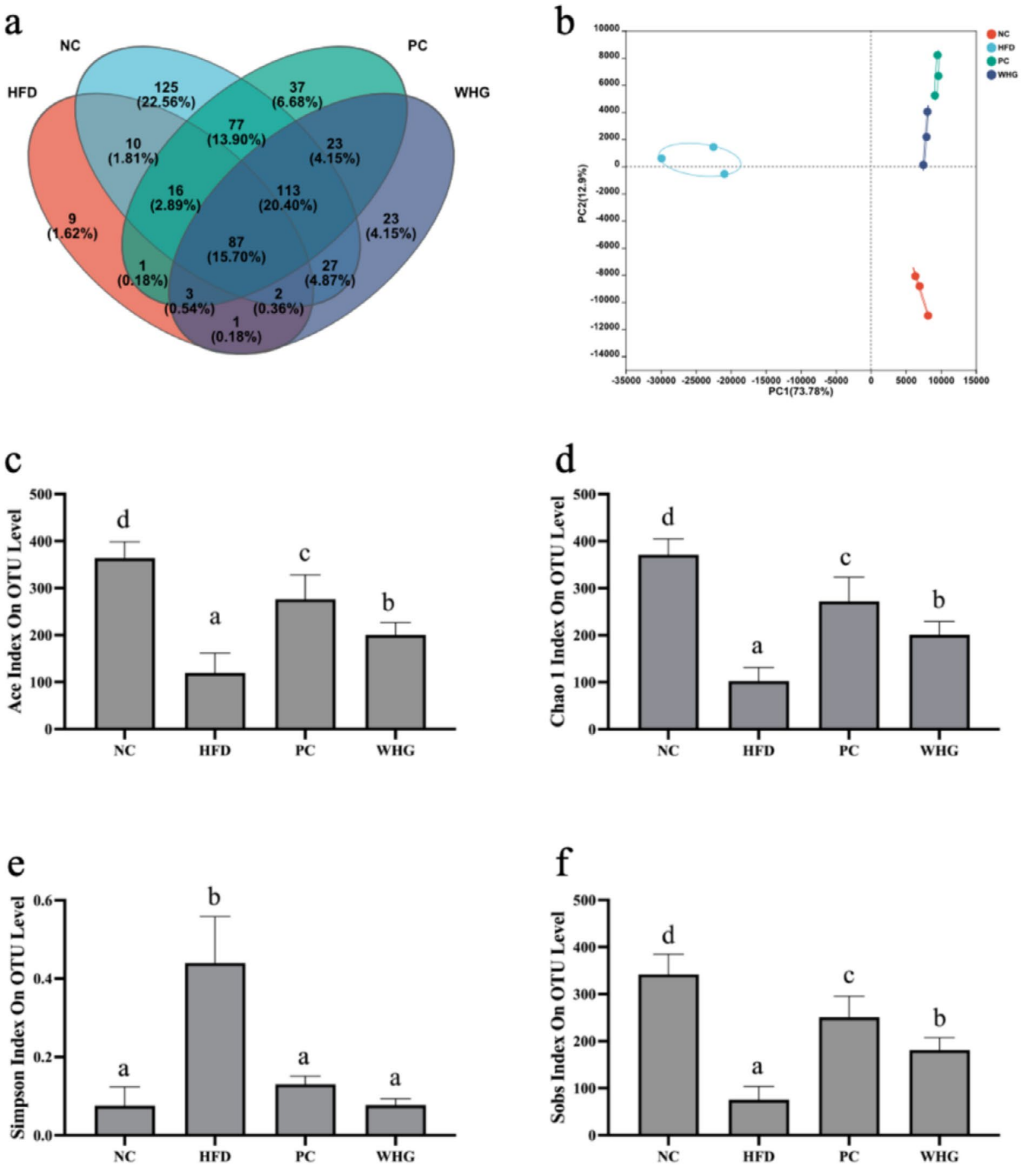
Effects of FMRP on Gut Microbiota in Mice (a) Venn diagram, (b) PCA analysis, (c) Ace index, (d) Chao1 index, (e) Simpson index, and (f) Sobs index. All data are expressed as mean ± standard deviation (*n* = 3). Different letters above the bars indicate statistically significant differences (*p* < 0.05), which also apply to the subsequent figures.

The effects of FMRP on gut microbiota diversity in mice are presented in Figure 5. The NC group had a total of 457 OTUs, while the HFD group, after consuming an hfd, had a total of 129 OTUs, with the two groups sharing 10 OTUs. Following intervention with FMRP, the NC group shared 77 OTUs with the PC group and 27 OTUs with the WHG group (Figure 5a). According to the PCA component analysis (Figure 5b), the NC group was distributed in the fourth quadrant, while the PC and WHG groups clustered in the first quadrant, closer to the NC group. This result is consistent with the Venn diagram, suggesting that the intervention effectively restored gut microbiota OTU changes induced by the hfd.

Additionally, the Ace index, Chao1 index, and Sobs in the HFD group were significantly lower than those in the NC group (*p* < 0.05). After intervention with the FMRP, these indices significantly increased (*p* < 0.05), indicating that FMRP restored gut microbiota abundance. In contrast, the Simpson index in the HFD group was significantly higher than that in the other groups (*p* < 0.05), indicating that obesity reduces gut microbiota diversity. The FMRP effectively inhibited this trend.

### 3.8 Effects of FMRP on the Composition of Gut Microbiota in Mice

**FIGURE 6:**
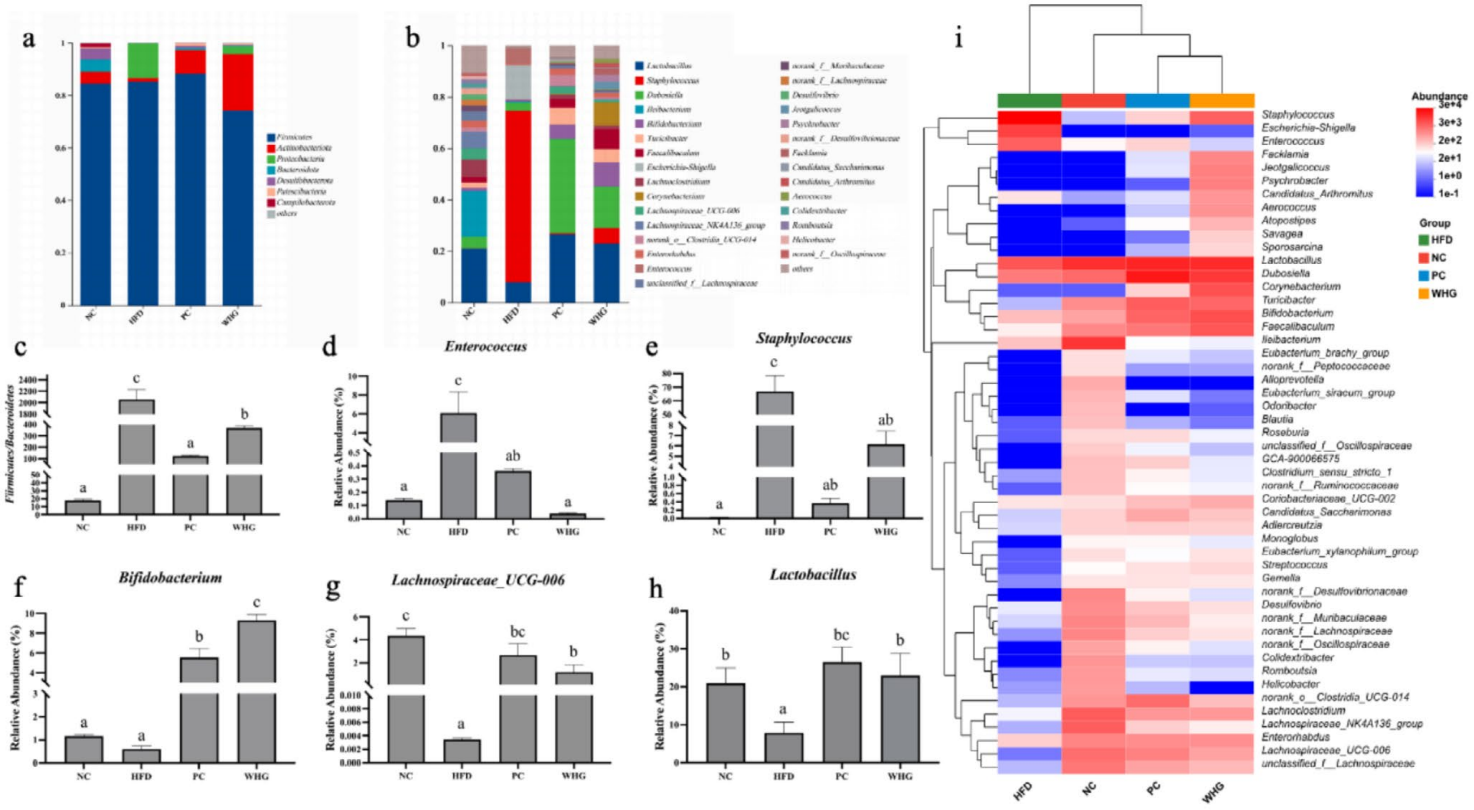
Effects of Meal Replacement on Gut Microbiota Structure in Mice (a) Phylum level, (b) Genus level, (c) F/B ratio, (d) *Enterococcus*, (e) *Staphylococcus*, (f) *Bifidobacterium*, (g) *Lachnospiraceae*, (h) *Lactobacillus*, and (i) Heatmap clustering analysis. Different letters indicate significant differences at the *p* < 0.05 level for each column.

The effects of FMRP on the gut microbiota composition in mice are illustrated in Figure 6. At the phylum level, *Firmicutes* was the dominant phylum in the gut microbiota of all groups. In addition, the NC group exhibited three other dominant phyla: *Bacteroidetes*, *Thermodesulfobacteria*, and *Actinobacteria*. Compared to the NC group, the HFD group showed an increase in the relative abundance of *Firmicutes* and *Proteobacteria*, while the relative abundances of *Bacteroidetes*, *Thermodesulfobacteria*, and *Actinobacteria* decreased. The intervention with FMRP reduced the relative abundance of *Firmicutes* and *Proteobacteria* (Figure 6a).

At the genus level, the dominant genera in the NC group included *Lactobacillus*, *Dubosiella*, *Ileibacterium*, *Lachnoclostridium*, and *Lachnospiraceae_NK4A136_group*. However, in the HFD group, *Staphylococcus* and *Escherichia–Shigella* dominated, accounting for over 80% of the relative abundance. After intervention, *Enterococcus* (Figure 6d) and *Staphylococcus* (Figure 6e) significantly decreased (*p* < 0.05), while the relative abundances of *Bifidobacterium* (Figure 6f), *Lachnospiraceae UCG‐006* (Figure 6g), and *Lactobacillus* (Figure 6h) significantly increased (*p* < 0.05). Additionally, a hfd significantly increased the F/B ratio in the gut microbiota (*p* < 0.05), while intervention with the FMRP significantly inhibited this trend (*p* < 0.05).

To more intuitively compare gut microbiota structure differences among the groups, clustering analysis was conducted at the genus level. The results showed that the HFD group formed a distinct cluster, while the NC, PC, and WHG groups clustered together, indicating that a hfd significantly alters gut microbiota composition, and the FMRP effectively restores the disrupted microbiota structure.

### 3.9 Correlation Analysis Between Obesity Traits and Gut Microbiota

**FIGURE 7:**
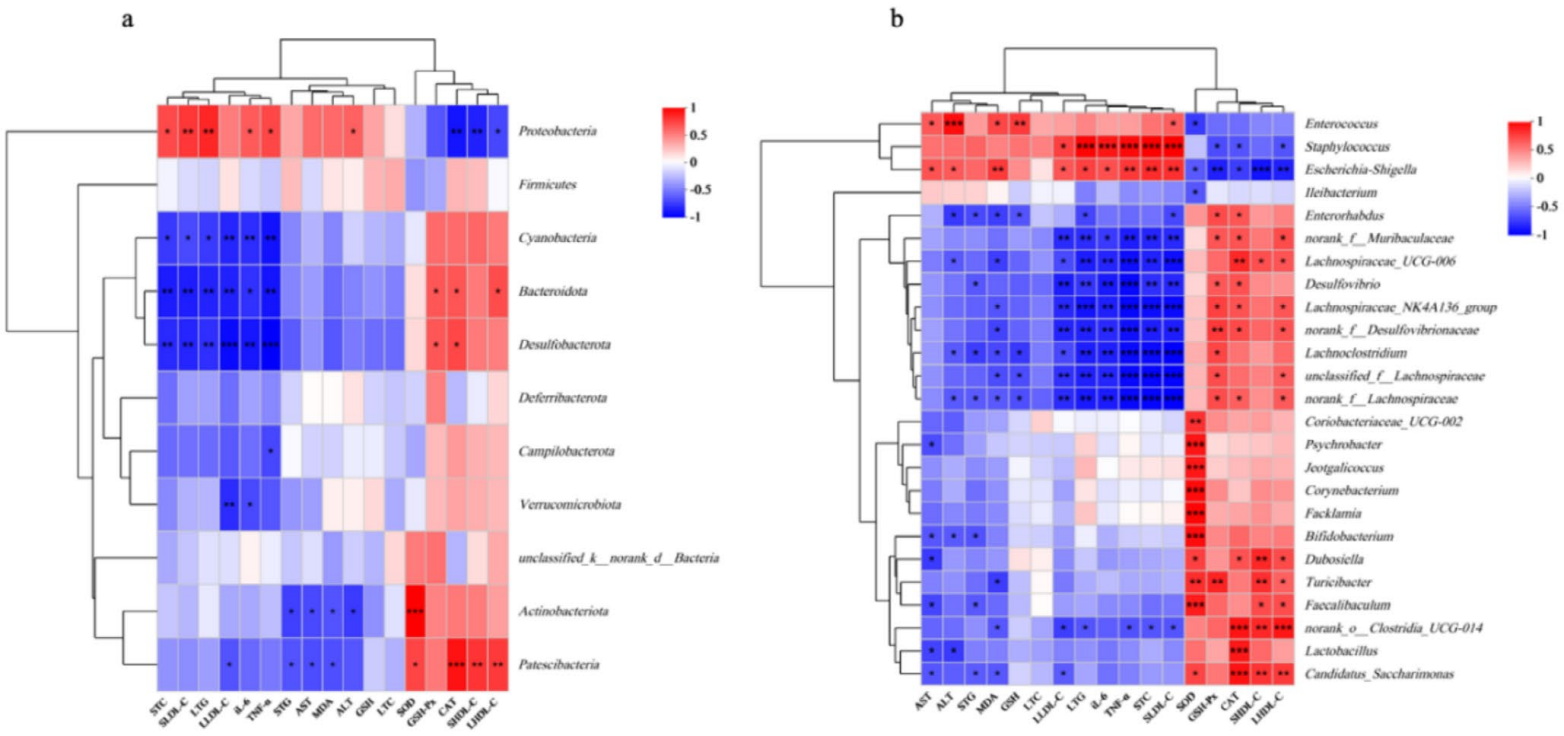
Correlation Analysis Between Obesity Traits and Gut Microbiota (a) Correlation between obesity traits and gut microbiota at the phylum level, and (b) Correlation between obesity traits and gut microbiota at the genus level. * indicates *p* < 0.05, ** indicates *p* < 0.01, and *** indicates *p* < 0.001.

The correlation between obesity traits and gut microbiota in mice was analyzed using Spearman correlation, including lipid levels, oxidative stress levels, and inflammation markers. The results of the analysis are presented in Figure 7. At the phylum level, *Proteobacteria* was positively correlated with body fat levels, while *Cyanobacteria*, *Desulfobacterota*, and other phyla were negatively correlated with body fat and inflammation levels. *Bacteroidota* and *Patescibacteria* were positively correlated with oxidative stress enzyme activity.

At the genus level, *Bifidobacterium*, *Lactobacillus*, and *Turicibacter* showed positive correlations with oxidative stress enzyme activity and negative correlations with liver damage. *Lachnospiraceae_UCG‐006*, *Desulfovibrio*, *Lachnoclostridium*, *unclassified_f_Lachnospiraceae*, and *norank_f_Lachnospiraceae* were negatively correlated with body fat and inflammation levels. In contrast, *Staphylococcus* and *Escherichia–Shigella* were positively correlated with body fat and inflammation levels. These findings suggest that differences in gut microbiota at both the genus and phylum levels are closely related to obesity, indicating that gut microbiota structure could serve as an important target for obesity characterization and treatment.

## 4 Discussion

This study assessed the weight loss and lipid‐lowering potential of an optimized FMRP with high‐antioxidant capacity in obese mice. It investigated the effects of various doses of the powder on the mice's physical characteristics, oxidative stress levels, and inflammation markers. By analyzing the correlation between changes in gut microbiota composition and the health status of the obese mice, the study offers preliminary insights into the potential link between the FMRP's effects on weight loss and lipid reduction and its modulation of the gut microbiota. In this study, the FMRP was primarily composed of figs, mulberries, yam, pumpkin, adzuki beans, black sesame, and brown rice. Figs are rich in functional compounds such as polyphenols and polysaccharides (Xu et al. 2024). Mulberries and adzuki beans are abundant sources of phenolic compounds, including flavonoids, anthocyanins, and phenolic acids (Martínez‐Alonso et al. 2022; Wen et al. 2019). Among these, polyphenols have been shown to enhance antioxidant capacity by increasing the activity of key enzymes such as SOD and GSH‐PX, activating the Nrf2 pathway, and inhibiting the NF‐κB signaling pathway (Lv et al. 2021). Additionally, polyphenols have been found to regulate lipid metabolism and adipogenesis through modulation of the mTOR signaling pathway (Cao et al. 2022). Furthermore, consumption of FMRP significantly reduced both food intake and the food efficiency ratio in mice (*p* < 0.05) and effectively suppressed hfd‐induced weight gain (Figure 1). This suggests that the FMRP enhances satiety, potentially lowering the secretion of appetite‐related factors (Esmaeili et al. 2022). The decrease in the food efficiency ratio further indicates that the powder reduces nutrient digestion and absorption in mice. These findings are consistent with previous research, which showed that dietary polyphenols in figs, mulberries, and adzuki beans stimulate the secretion of satiety hormones such as cholecystokinin (CCK) and glucagon‐like peptide‐1 (GLP‐1), helping to regulate appetite and food intake (Reis et al. 2021).

The liver is a key organ for lipid metabolism and storage, where fatty acids and TG are synthesized and energy is stored. Additionally, the liver synthesizes and secretes various lipoproteins to transport lipids throughout the body, helping to maintain overall energy balance. However, excessive lipid intake can disrupt this balance, leading to elevated blood lipid levels. In this study, obese mice showed significantly increased levels of TC and TG (*p* < 0.05) (Figures 2a,b and 3c,d). Prolonged lipid accumulation resulted in an increase in liver weight and significantly enlarged lipid vacuoles in the liver, which led to a marked rise in ALT and AST levels (*p* < 0.05) (Figure 2e,f), indicating impaired liver function in the HFD group. HDL‐C plays a crucial role in transporting cholesterol to the liver for metabolism and excretion, effectively reducing TC and TG levels (Marseglia et al. 2015). After consuming the FMRP, HDL‐C levels in both the liver and blood were significantly elevated (*p* < 0.05) (Figures 2d and 3f), while TC and TG levels in the liver and blood, along with ALT and AST levels in the blood, were significantly reduced (*p* < 0.05). These results suggest that the FMRP has the potential to restore lipid metabolism and regulate disturbances in blood and liver lipid levels caused by obesity.

Obesity leads to an accumulation of adipose tissue, which generates significant amounts of ROS (Pérez‐Torres et al. 2021). Research has indicated that the onset of obesity results in a reduction in the activity of key antioxidant enzymes, including SOD, CAT, and GSH‐PX, thus inducing oxidative stress. In addition, obesity exacerbates inflammation in adipose tissue, leading to an increased secretion of pro‐inflammatory factors (Talpo et al. 2022). A similar trend was observed in this study, where obesity reduced the activities of oxidative stress enzymes SOD, CAT, and GSH‐PX, while increasing oxidative damage markers such as MDA and GSH, as well as inflammatory factors IL‐6 and TNF‐α. Intervention with FMRP significantly enhanced enzyme activity, reducing oxidative stress damage and alleviating the inflammatory response. Previous studies have shown that compounds such as quercetin, catechin, and cyanidin in figs possess anti‐inflammatory properties (Zhang, Peng et al. 2024), while mulberry polysaccharides, anthocyanins, and phenolic acids have demonstrated the ability to reduce oxidative stress damage (Lou et al. 2024; Wang and Huang 2024).

Current research shows that gut microbiota is closely linked to obesity, making it an important target for obesity intervention (Ley et al. 2005). This finding is consistent with the results of previous studies, which identified significant correlations between gut microbiota composition, both at the phylum and genus levels, and obesity, oxidative stress, and inflammation. Specifically, at the phylum level, *Bacteroidota* and *Patescibacteria* were positively associated with oxidative stress enzyme activity in mice. At the genus level, *Lachnospiraceae_UCG‐006*, *Lachnoclostridium*, unclassified *Lachnospiraceae* species, and *norank_f_Lachnospiraceae* were negatively correlated with body fat and inflammation levels. Moreover, previous research has shown that a hfd significantly increases the F/B ratio in the gut microbiota (Chen et al. 2018). In this study, intervention with the FMRP significantly inhibited this trend (*p* < 0.05). Additionally, the FMRP effectively restored the gut microbiota diversity, which had been reduced by obesity (Figure 5). At the genus level, the FMRP also modulated key bacterial species such as *Lactobacillus* (Figure 6h) and *Bifidobacterium* (Figure 6f). Previous studies have shown that *Bifidobacterium* ferments dietary fibers to produce short‐chain fatty acids (SCFAs), such as acetate and propionate, which improve gut barrier function, reduce intestinal permeability, and suppress inflammation. Increased *Bifidobacterium* levels are also associated with reduced serum cholesterol and TG, contributing to better metabolic health. Similarly, *Lactobacillus* promotes lipid metabolism by modulating bile acid activity, reducing fat accumulation, and improving energy balance. The bioactive compounds in FMRP, including polyphenols and polysaccharides, support the growth of these beneficial bacteria, enhance SCFA production, and regulate key metabolic pathways. These findings suggest that FMRP improves gut microbiota composition, providing a foundation for its anti‐obesity and metabolic benefits (Chen et al. 2018; Zhao et al. 2021). Furthermore, heatmap‐clustering analysis confirms that the FMRP intervention effectively restored the gut microbiota structure that was disrupted by obesity.

## 5 Conclusion

In conclusion, FMRP has the potential to alleviate obesity by effectively regulating oxidative stress and modulating immune responses through the correction of gut microbiota imbalances caused by a hfd. This study offers valuable insights for increasing the added value of fig processing and for the development of fig‐based functional foods.

## Author Contributions

Lixia Xiao designed the study; Mingze Xu, Longfei Zhang, and Xiaoxiao Liu performed the mice experiment; Mingze Xu analyzed and interpreted the data; Mingze Xu and Longfei Zhang wrote the manuscript; Yigu Tian and Bingkui Wang edited the manuscript and conducted the publishing process; Wenlong Ma and Tianzhu Guan gave the technical support and conceptual advice. Hengxian Qu and Dawei Chen guided and supervised us during the whole manuscript process. All authors have read and approved the final manuscript.

## Conflicts of Interest

The authors declare no conflicts of interest.

## Supporting information


**Data S1**.

## Data Availability

Supporting Information associated with this article can be found, in the online version.
