# Supplementary material for: Fig Meal Replacement Powder Ameliorates Obesity, Oxidative Stress and Intestinal Microbiota in Mice Fed With High‐Fat Diet
Source: Food Sci Nutr. 2025 Apr 20;13(4):e70104. doi: 10.1002/fsn3.70104 (PMC12009752; doi:10.1002/fsn3.70104)
Supplement: Supplementary file 1 — Data S1. [file FSN3-13-e70104-s001.docx]

Standard Curves for DPPH, ABTS Radical Scavenging Capacity, and Fe²⁺ Scavenging Capacit

Figure S1 illustrates that the standard curves for DPPH, ABTS radical scavenging capacity, and Fe²⁺ scavenging capacity all demonstrate strong linearity. The Trolox concentration range for the DPPH standard curve was 5 μg/mL to 25 μg/mL, while the range for the ABTS and FRAP standard curves was 0.15 mM to 1.5 mM. The correlation coefficients (R²) for the DPPH, ABTS, and FRAP standard curves were 0.999, 0.9992, and 0.9998, respectively.


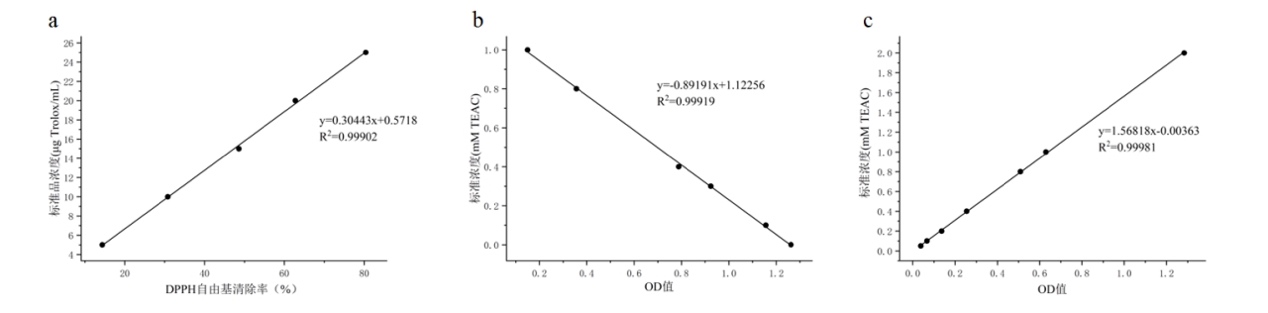


Figure S1. Standard curves for DPPH, ABTS and FRAP radical scavenging capacity (a) Standard curve for DPPH radical scavenging capacity, (b) Standard curve for ABTS radical scavenging capacity, and (c) Standard curve for FRAP radical scavenging capacity.
